# Evolution of nuptial gifts and its coevolutionary dynamics with male-like persistence traits of females for multiple mating

**DOI:** 10.1186/s12862-021-01901-x

**Published:** 2021-09-05

**Authors:** Yoshitaka Kamimura, Kazunori Yoshizawa, Charles Lienhard, Rodrigo L. Ferreira, Jun Abe

**Affiliations:** 1 Department of Biology, Keio University, Yokohama, 223-8521 Japan; 2 Systematic Entomology, School of Agriculture, Hokkaido University, Sapporo, 060-8589 Japan; 3 Geneva Natural History Museum, CP 6434, 1211 Geneva 6, Switzerland; 4 Biology Department, Federal University of Lavras, Lavras, MG 37200-000 Brazil; 5 Faculty of Liberal Arts, Meijigakuin University, Yokohama, Japan

**Keywords:** Nuptial gift, Paternity determination mechanism, Coevolution, Female persistence trait, Female penis, Sex role reversal

## Abstract

**Background:**

Many male animals donate nutritive materials during courtship or mating to their female mates. Donation of large-sized gifts, though costly to prepare, can result in increased sperm transfer during mating and delayed remating of the females, resulting in higher paternity. Nuptial gifting sometimes causes severe female-female competition for obtaining gifts (i.e., sex-role reversal in mate competition) and selection on females to increase their mating rate, changing the intensity of sperm competition and the resultant paternity gains. We built a theoretical model to simulate such coevolutionary feedbacks between nuptial gift size (male trait) and propensity for multiple mating (female trait). Donation of nuptial gifts sometimes causes development of female persistence trait for gift acquisition. We also analyzed the causes and consequences of this type of traits, taking double receptacles for nutritious seminal gifts, which are known to occur in an insect group with a “female penis” (*Neotrogla* spp.), as an illustrative example.

**Results:**

Our individual-based simulations demonstrated that female-female competition for male-derived nutrients always occur when the environment is oligotrophic and mating costs are low for females. However, a positive correlation between donated gift size and the resultant paternity gain was a requisite for the co-occurrence of large gifts and females’ competitive multiple mating for the gifts. When gift donation satisfied female demands and thus resulted in monandry, exaggeration of nuptial gift size also occurred under the assumption that the last male monopolizes paternity. The evolution of double slots for gift acquisition and digestion (female persistence trait) always occurred when males could not satisfy the demands of females for gifts. However, through coevolutionary reduction in male gift size, fixation of this trait in a population drastically reduced the average female fitness.

**Conclusion:**

Sperm usage patterns, which have rarely been examined for animals with nuptial gifts, can be a critical factor for determining the extent of exaggeration in nuptial gifting. Sex-role reversals in mate competition, as a result of donation of nuptial gifts from males to females, can involve the evolution of male-like, persistent traits in females that reduce population productivity, as is the case with persistence traits in males.

**Supplementary Information:**

The online version contains supplementary material available at 10.1186/s12862-021-01901-x.

## Background

Nuptial gifts, any non-gametic materials transferred from one sex (usually male) to another during courtship and mating that improve donor fitness, are widely observed in many groups of animals, such as insects, arachnids, molluscs, amphibians, birds, and mammals including humans [1, 2]. In some cases, male-derived “gifts” can be detrimental to female recipients, as with the love darts of land snails and anti-aphrodisiac seminal peptides of *Drosophila* fruit flies, both of which mitigate the intensity of sperm competition in female sperm storage organs [3, 4]. However, in many cases, males supply and benefit females with nutritious materials such as prey items they have collected or voluminous secretions from male internal/external glands [1, 5].

Although this phenomenon is widely observed in the animal kingdom, there is continuing debate on its primary function [6–13]. Since females of some animals accept mating (and sperm transfer) only while consuming a gift, males may donate the gift to obtain mating opportunities (i.e., mating effort hypothesis). In addition to nourishing female recipients, nutrition from nuptial gifts can be passed to offspring sired by the male donor, and thus can function as paternal investment (i.e., paternal investment hypothesis: [9, 11, 13]). Recent studies suggest that these two hypotheses are not mutually exclusive and are difficult to discriminate, because male-derived nutrients cannot be properly allocated to the offspring of respective donors without a positive correlation between the extent of mating effort by males and their paternity success [1].

Regardless of the ultimate benefits for males, a large nutritious gift is costly for the male to obtain or produce (reviewed in [5]), although it may be more attractive to females or may enable transfer of more sperm [1, 14, 15]. Accordingly, donation of exaggerated nuptial gifts may cause partial reversals in sex roles under certain circumstances like limited food. Thus, in contrast to conventional animals where males more actively seek mating opportunities, females compete for multiple mating opportunities to obtain more nuptial gifts in animals with sex-reversed mate competition [13, 16–18]. Increased polyandry can result in more severe sperm competition, changing the cost–benefit balance for males of preparing nuptial gifts. Although several models have been proposed to date for elucidating the evolution of nuptial gifts [10, 19, 20], they did not encompass these possible coevolutionary feedbacks between the sexes for the trading of gifts. The patterns of female sperm storage and use, which determine the benefits in paternity gain of donating a given size of gift, are possible pivotal factors for shaping the coevolutionary dynamics in the trading of nuptial gifts. However, no theoretical studies have explicitly incorporated these aspects so far.

To analyze the coevolutionary process between male-derived gifts and female propensity for multiple matings for the first time, we develop an individual-based model in this study incorporating post-copulatory sexual selection. The effects of the environmental/ecological factors (mating costs for females and resource availability) and sperm usage patterns on the coevolutionary process are also evaluated. Since male donors cannot exactly control the size of prey items, we model the evolution of seminal gift size, which can be treated as an evolvable trait (e.g., [21]).

### Evolution of female morphology for gift-acquisition

Donation of nuptial gifts sometimes causes development of female persistence trait for gift acquisition. As an example of this type of traits, the members of *Neotrogla* (Insecta: Psocodea: Prinoglarididae: Sensitibillini) are of special interest. In all four known species of this genus, females possess an evolutionarily novel penis-like structure, termed a gynosome [22]. During copulation which lasts for a long period (41–73 h in *N. curvata*) with the female positioned above the male, a female inserts this “female penis” to the vagina-like male genitalia [22]. During this, seminal fluid which contains voluminous and potentially nutritious seminal substances is transferred to the female through the gynosome and an elongated duct (spermathecal duct) [22]. Then, a voluminous ejaculate forms a gigantic, bottle-shaped capsule (spermatophore) in the female body [22, 23]. The gynosome is ornamented with species-specific lobes and/or spine bundles, which are accommodated in specialized pouches of the male genital cavity during copulation [22]. Since they live in dry, nutritionally poor caves in Brazil, the gynosome likely represents a female adaptation for unwilling male mates to better grasp them and to exploit seminal gifts, though no physical damage has been detected in male “vagina”, [22, 24]. Although female-female competition for seminal gifts has not been directly observed for *Neotrogla* spp., females of a related species with similar spermatophores (Psocodea: Trogiidae: *Lepinotus*) compete for access to males [25–27].

Moreover, females of *Neotrogla* and those of related genera of the tribe Sensitibillini (*Afrotrogla* and *Sensitibilla*) have also developed a specialized structure, termed a spermathecal plate, in their sperm storage organ (spermatheca). In *Neotrogla* (and possibly also in *Afrotrogla* and *Sensitibilla*), this evolutionarily novel organ is equipped with twin slots that enable retention and digestion of two seminal gifts simultaneously [22–24, 28]. By contrast, females of related groups with only a single slot for nuptial gift can accept another mating only after digestion of the content of a spermatophore received at the preceding mating (for example, in *Lepinotus* [26]). Given that *Neotrogla* females mate multiply as evidenced by up to two full and nine emptied spermatophore capsules in their spermatheca [22], the spermathecal plate of this genus can be considered another female persistence trait for competitively obtaining male-derived gifts in rapid succession. As an extension of our basic model, we also analyzed the causes and consequences of the evolution of this female persistence trait, i.e., twin slots for gift reception and digestion, in coevolutionary dynamics between male gift size and female propensity for multiple mating.

### Outline of the simulation methods

**Fig. 1:**
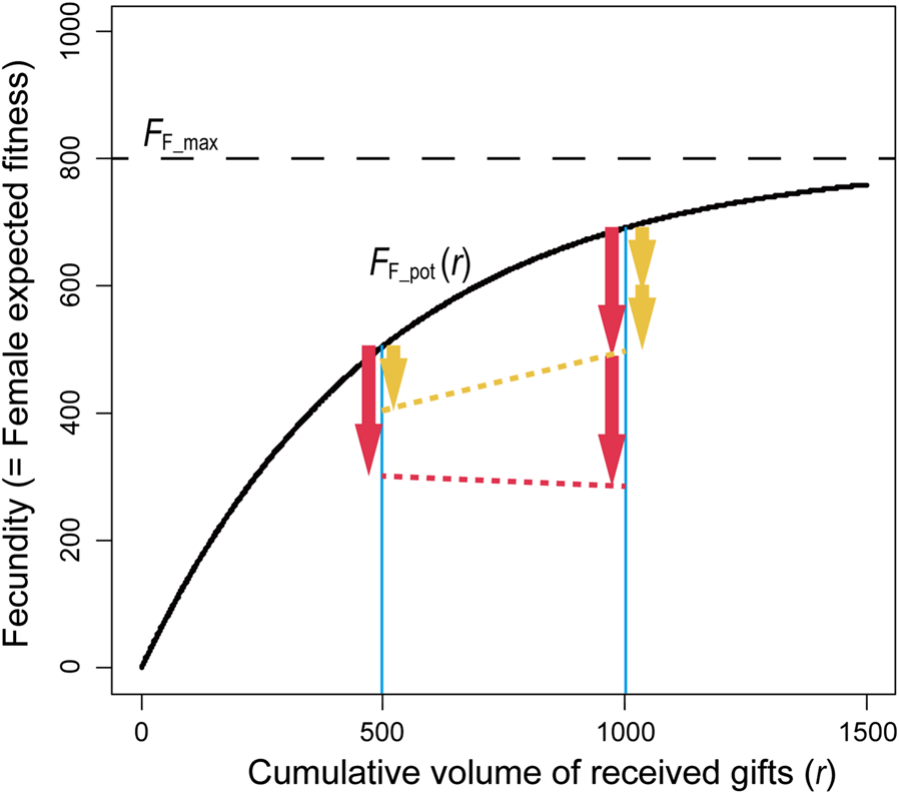
Female fecundity as a saturating function of cumulative seminal nutrients from males (*r*), based on the assumption that a given volume of nuptial gift will increase the female’s fitness more effectively when the female is starving. When males provide a seminal gift of size 500 (*V* = 500), females that mated once should seek another mating opportunity when the cost of mating is low (orange arrows, *c* = 100 per mating), but not under a higher cost (red arrows, *c* = 200). See Eqs. (1) and (2) in the main text for the details

We simulated the coevolutionary dynamics between two traits with sex-specific expression in a population of sexually-reproducing, diploid organisms of a constant size (500 females plus 500 males): the size (volume) of nutritive seminal gifts produced by males (*V*) and female propensity for multiple mating to obtain gifts (the number of additional matings: *M*). Each of these traits were assumed to be determined by alleles on a single locus (*v1* and *v2*, and *m1* and *m2*), which were continuously variable and subject to recurrent mutations. No linkage was assumed between these two loci. We made only two basic assumptions on the effects of these traits on male and female fitness: (1) the offspring number of a female (fecundity) is a saturation function (Fig. 1) of the cumulative volume of seminal gifts (*r*) received in all of her previous matings, reduced by mating costs (*c*) multiplied by the number of realized matings (*M*_*R*_), and (2) reception of a gift, which occupies a slot for its digestion, delays subsequent matings of the female, in a manner proportional to the gift size. Thus, given that males acquire a limited amount of resources for gift production (*R*), males that produce large-sized gifts can reduce the probability of remating of the female mates while increasing their fecundity, but suffer a reduced number of possible matings (*N* ≈ *R*/*V*; a size-number trade-off). To avoid unnecessary complexities, any precopulatory behaviors, such as male-male combat over mates or female choice on gift size or males, were not incorporated.

**Fig. 2:**
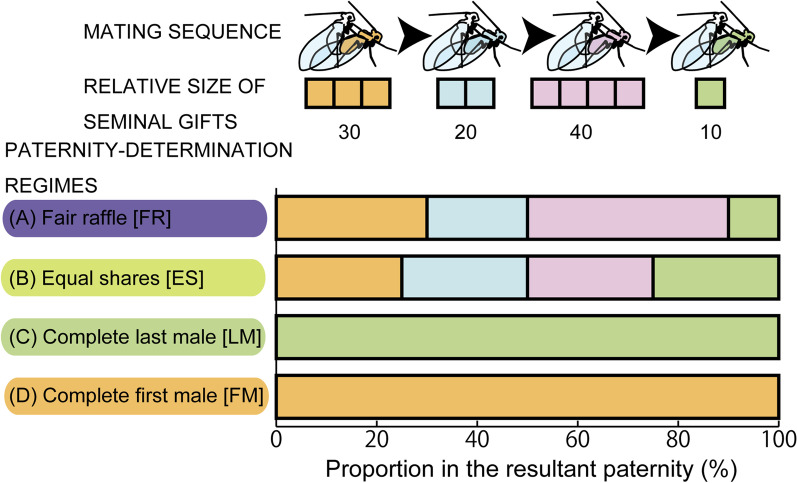
A scheme of four different regimes of the relationships between seminal gift size and the resultant paternity. Note that *Neotrogla* spp. shows a female-above mating posture similar to many other species of Psocodea

Since sperm storage/use patterns of multiply-mated females are largely unknown for animals with donation of nuptial gifts [1], we examined four different regimes for the relationship between the nuptial gift size and the resultant paternity gain in this study: fair raffle (FR), equal shares (ES), complete last male (LM), and complete first male (FM) (Fig. 2, Table 1). Simulations were repeated for each of these four paternity-determination schemes, in combination with variable resource availability to males (*R*) and mating costs for females (*c*).

**Table 1:** Summary of notations and abbreviations used in this study

Symbol	Definition and designated values
Model parameters	
* v1, v2*	Genotypic values of male seminal gift size from male and female parents, respectively. Initial values were given as a normal distribution (mean ± SD = 20 ± 40) with truncation (≥ 20)
* V*	Phenotypic value of male seminal gift size, calculated as (*v1* + *v2*)/2
* V*_*last*_	Seminal volume of the last mating of a focal male (Eq. 5)
* m1, m2*	Genotypic values of female acceptable number of matings from male and female parents, respectively. Initial values were given as a normal distribution (mean ± SD = 0.5 ± 2) with truncation (≥ 0)
* M*	Phenotypic value of female acceptable number of additional matings (= propensity for multiple mating), calculated as (*m1* + *m2*)/2
* M*_*R*_	Realized number of matings by females
* M*_*O*_	Optimal number of matings by females, given by solving Eq. 2 with the mean *V*
* 2s*	Dominant gene for producing twin slots for receiving gifts
* 1s*	Recessive gene for producing a single slot for receiving gifts
* 2S*	Females of the genotype *2s*/*2s* or *2s*/*1s*, having twin slots for receiving gifts
* 1S*	Females of the genotype *1s*/*1s*, having a single slot for receiving gifts
* R*	Mean total volume of resource for producing seminal gifts, ranged from 400 (oligotrophic) to 1300 (eutrophic) by an increment of 100. Each male has a resource budget extracted from a truncated normal distribution mean ± SD = *R* ± 0.2*R* (> 0)
* N*	Possible number of matings for a focal male, given as *R*/*V*, rounded up to the nearest integer
* n*	Realized number of matings for a focal male
* r*	Cumulative volume of gifts received by a female
* b*	Fertilization efficiency (Eq. 1; Fig. 1), given as a constant (*b* = 0.002)
* c*	Female mating cost (per mating), ranged from 5 to 95 by an increment of 10 (Eq. 2)
* F*_F_max_	Maximum female reproductive output (Eq. 1), given as a constant (*F*_F_max_ = 800)
* F*_F_pot_(*r*)	Maximum fecundity of a female, as a function of *b*, *F*_F_max_ and *r*, before reduction by mating costs (Eq. 1)
* F*_F_(*r, c, M*_*R*_)	Realized fecundity (= expected fitness) of a female, as a function of *b*, *F*_F_max_, *r*, *M*_*R*_, and *c* (Eq. 2)
* P*	Paternity share of a male in the offspring of a female mate
* F*_M_(*V*)	Expected fitness of a male defined by Eq. 3
Paternity determination regimes	
FR	Paternity is determined by fair raffle with respect to the relative gift size received by a focal female
ES	Paternity is equally shared by all male mates
LM	The last male monopolizes paternity
FM	The first male monopolizes paternity
Simulation modes	
CON	Control simulation runs with neither invasion of *2S* females nor doubling of female number
DS	One *2S* female per generation was introduced from the1000th generation and beyond
DF	Number of females were doubled at the 1000th generation and beyond (i.e., 1000 females for 500 males)

## Results

### Coevolution of nuptial gift size and female multiple mating under different paternity-determination regimes

**Fig. 3:**
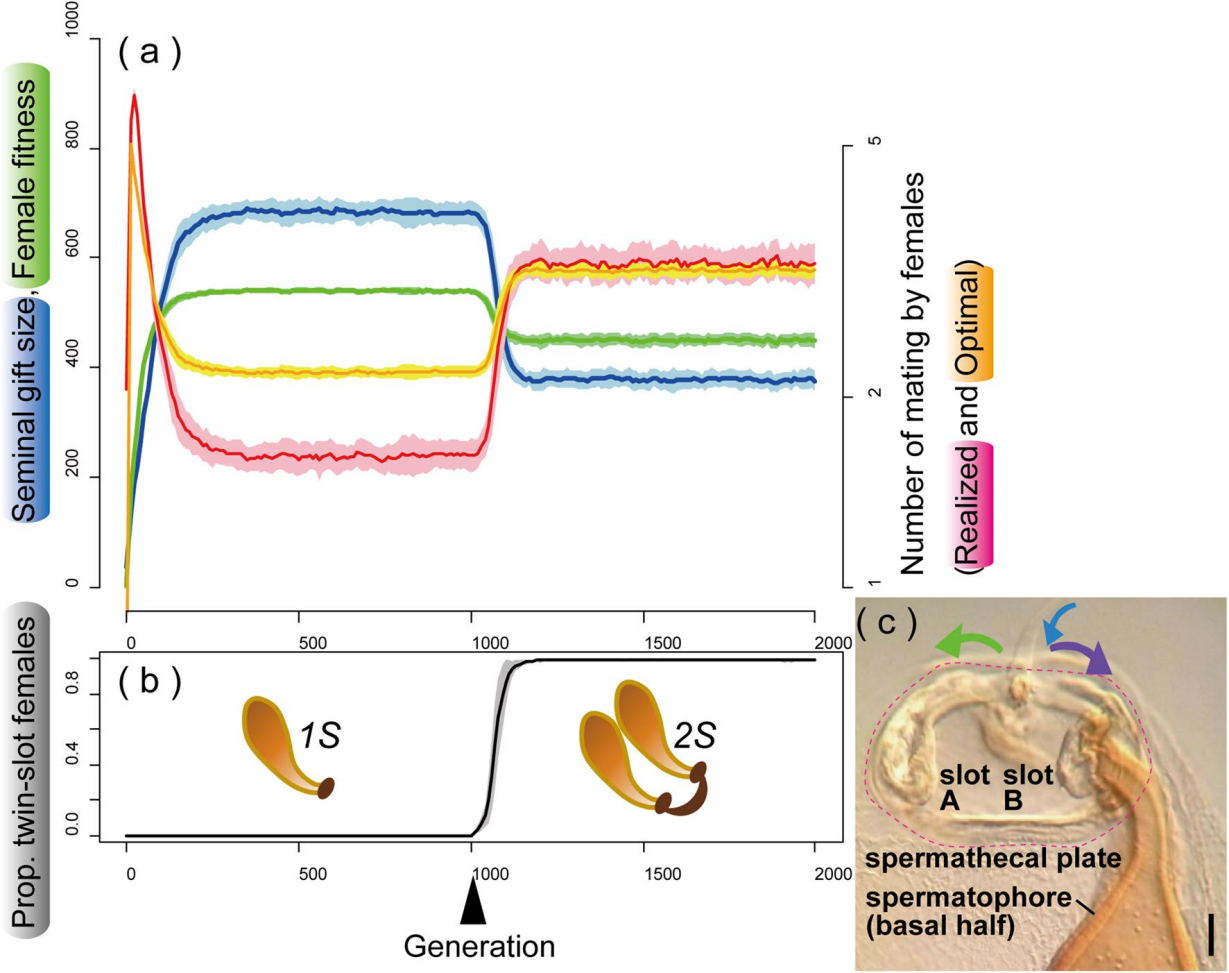
**a** An example of the coevolutionary dynamics observed between the male seminal gift size (*V*, blue) and the optimal number of matings for females (*M*_*O*_, red), together with changes in the realized number of matings by females (*M*_*R*_, orange) and female fitness (*F*, green). **b** Changes in the proportion of females with twin slots (*2S*), which started to invade the population at the 1000th generation (the black arrowhead), with schematics of *1S* and *2S* states. Solid lines and shaded areas of respective lighter colors show the mean ± SD for 40 runs under the FR (fair raffle) regime (*R* = 800, *c* = 55). **c** Spermathecal plate (delineated by red dashed line) of *Neotrogla truncata* with a spermatophore. A female can actively control the direction of seminal flow (blue arrow), either to slot A (green arrow) or to slot B (purple arrow). Scale bar: 50 µm

**Fig. 4:**
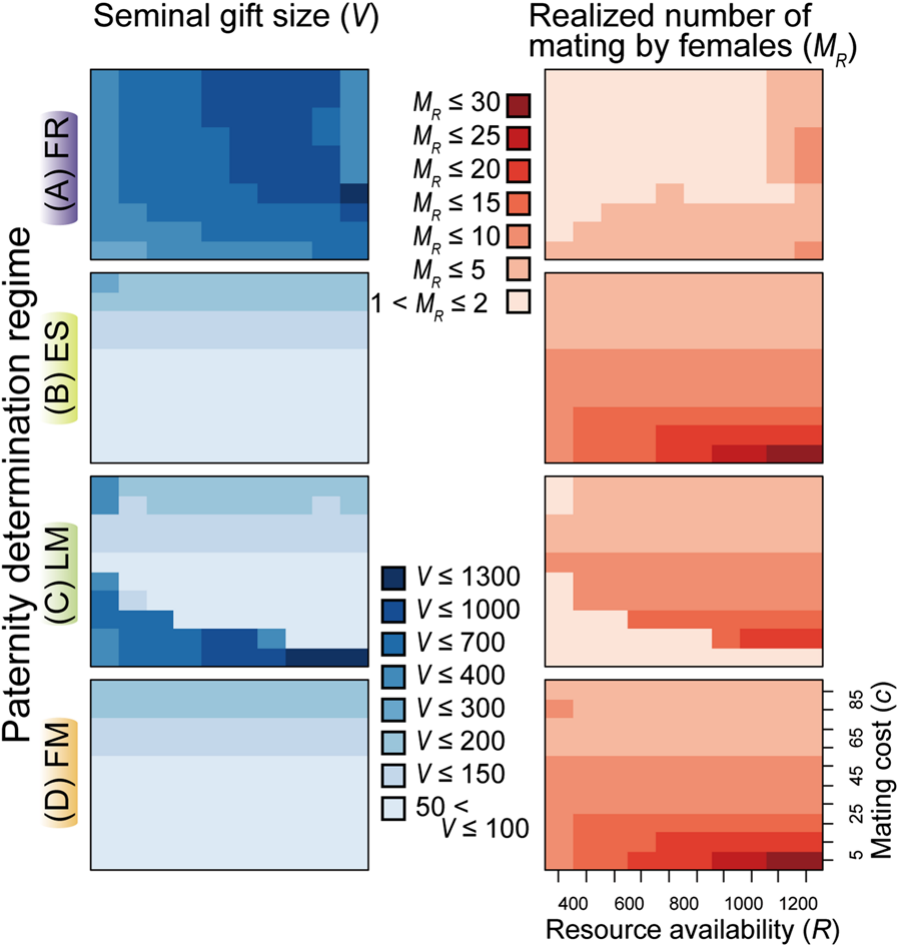
Average male seminal gift size (*V*, light blue backgrounds) and the realized number of matings by females (*M*_*R*_, pink backgrounds) at the 2000th generation observed in the control runs (CON) of four different paternity-determination regimes (**A** FR [fair raffle]; **B** ES [equal shares]; **C** LM [complete last male]; and **D** FM [complete first male])

Our simulations revealed notable effects of the pattern of paternity determination on the evolution of gigantic seminal gifts and female propensity for multiple mating. Under a given set of parameters, the male seminal gift size and female propensity for multiple mating rapidly converged to an equilibrium, usually before the 300th generation (the left half of Fig. 3a, b; Additional file 1). Only when females used sperm from each male mate for fertilization of eggs proportionally to the nuptial gift size donated (the FR regime) did males evolve a large-sized gift compared with their lifetime resource budget under a wide range of parameter sets (male resource budget [*R*] and cost of mating for females [*c*]: Fig. 4A).

**Fig. 5:**
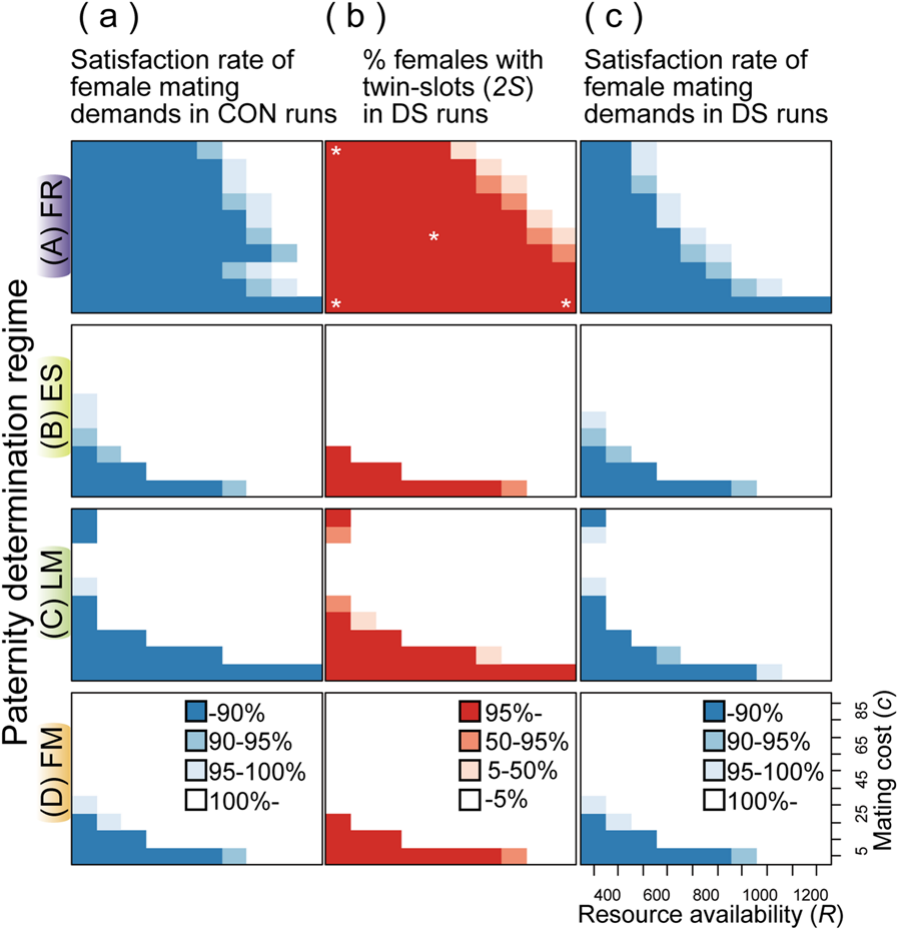
Average satisfaction rate (*M*_*R*_/*M*_*O*_) of female mating demands in CON [control]** (a)** and DS [twin-slots invasion] **(c)** runs, and proportion of *2S* females **(b)** in DS runs at the 2000th generation (**A** FR [fair raffle]; **B** ES [equal shares]; **C** LM [complete last male]; and **D** FM [complete first male]). The asterisks in **A-b** indicate the parameter sets examined in Fig. 6

Under the situations assumed in our simulation, dividing the limited resource (*R*) into small-sized gifts could increase the mating opportunities of males, while a large-sized nuptial gift could afford males two types of paternity benefits, namely: (1) siring more offspring than males who gave a smaller gift to the same mate; and (2) eliminating the probability of remating by the female as a post-copulatory guard against remating. Because the former type of benefit (siring more offspring) occurs only under the assumption of the FR regime, this can explain the observed prevalence of the “fewer large” strategy over “many small” in this regime. Accordingly, females evolve the propensity to mate multiply for these attractive, large-sized gifts when available, but males generally cannot satisfy the inflated females’ demands (Figs. 4A, 5A). Exceptions are when the mating cost is extremely large (high *c*) and the environment is eutrophic (high *R*, the upper-right corners of Figs. 4A, 5A). Large mating costs for females reduces the attractiveness of a given size of gifts, making a few matings optimal for females. The latter condition can shift male strategies to “many small” for seeking many (virgin) females, rendering their gifts more unattractive to females.

When the paternity was equally assigned to all mates of a female (ES) or was monopolized by the first male (FM), the coevolutionary patterns were quite similar indicating almost identical sexual selection pressures under these two regimes. Males generally did not evolve a large-sized nuptial gift (Fig. 4B, D). As discussed above, this can be attributed to the lack of paternity benefits proportional to the donated gift size in these regimes. To collect these small gifts from many males, females mated more frequently than under the FR regime, especially when mating cost was low and the habitat was eutrophic (Fig. 4B, D). By increasing the acceptable number of matings, females usually achieved their optimal number of matings (Fig. 5B, D). An exception is the condition with very small mating costs and oligotrophic environments (the lower-left corners of Fig. 4B, D), where additional mating is always beneficial even for small-sized gifts.

When the last male monopolized the paternity (LM), males generally evolved larger-sized gifts compared to the FM (complete first male) and ES (equal shares) paternity-determination regimes (Fig. 4C). To be the last mate, we can envisage two different strategies: preparing many small gifts increases the opportunities for being the last mate by chance (as in the FM and ES regimes), while provision of a large-sized gift is advantageous because it reduces the probability of remating by the female. The latter strategy must be especially effective when females tend to seek additional mating opportunities because of low mating cost. Thus, the observed large gifts when *c* is low (the lower areas of Fig. 4C) can be a countermeasure of males for preventing frequent remating of their mates. When mating is more costly for females, it is less necessary for males to prepare large gifts, and the small-sized gifts reinforce the females’ reluctancy for further mating. Accordingly, for high *c* values, females satisfied their mating demands when the habitat was eutrophic (high *R*, Fig. 5C). An important exception is the extremely oligotrophic environments (*R* = 400) with extremely high cost of mating (*c* = 85–95) (the upper-left corners of Figs. 4, 5). Under this combination of parameters, males also evolve comparatively larger gifts under the ES and FM regimes, even though it does not result in overly high mating demands of females that males cannot satisfy. Given extremely high mating costs for females, males experience a low risk of sperm competition. In addition, if males have extremely limited resources for preparing gifts, premating male-male competition should be also less severe. Accordingly, males likely shift toward providing the monandrous female as much as their resource budget allows.

In our simulation, the genetic correlation between seminal gift size ([*v1* + *v2*]/2) and female mating propensity ([*m1* + *m2*]/2), calculated for a pooled population of male and female individuals, was negligibly low (near zero), and thus elimination of genetic covariance by shuffling paternal identity did not change the results (Additional file 2).

### The evolution of twin-slots and its effects on the coevolutionary processes

We also tested the evolvability and stability of another female trait, that is, the twin-slot state (*2S*) for receiving and digesting two seminal gifts simultaneously, as well as its effects on the evolution of the other two traits (male *V* and female *M*). For simplicity, we assumed that this trait is controlled by a single locus, which is independent of the *v* and *m* loci, with two alleles: *2s*, a gene for the twin-slot state, and *1s*, a gene for the single-slot state, where the former was assumed to be dominant over the latter. The populations were initiated with single-slot females (*1S*, *1s*/*1s* homozygotes). Then, *2s* genes were allowed to invade populations at the mid (the 1000th generation) of 2000-generation simulation runs and beyond (one spontaneous mutation in each generation), together with recurrent invasion of single-slot mutants (a female individual of *1s*/*1s* homozygote per generation) to check the evolutionary stability of the twin-slot state (*2S*). Abe and Kamimura [29] demonstrated that female-biased sex ratios promote the production of smaller ejaculate packages by males in order to mate with more females. Doubling the slot number can have a similar effect on male ejaculate allocation. Thus, for comparison to this invasion experiment (doubling-slot [DS] runs), the number of females was doubled, resulting in male:female = 1:2, from the mid (the 1000th generation) of simulation runs and beyond in “doubling females [DF]” runs. Neither the numbers of slots nor females were doubled throughout the 2000-generation runs in the control runs (CON: Table 1).

Figure 5 clearly shows that *2S* females successfully invaded into a population when females could not satisfy their required number of matings on average (Fig. 5). Such a condition occurs (1) when mating costs for females are extremely low and the environment is oligotrophic regardless of the paternity determination regimes, or (2) when males evolve large-sized gifts, reception of which is beneficial for females and outweighs the associated mating costs (Fig. 4A, C).

**Fig. 6:**
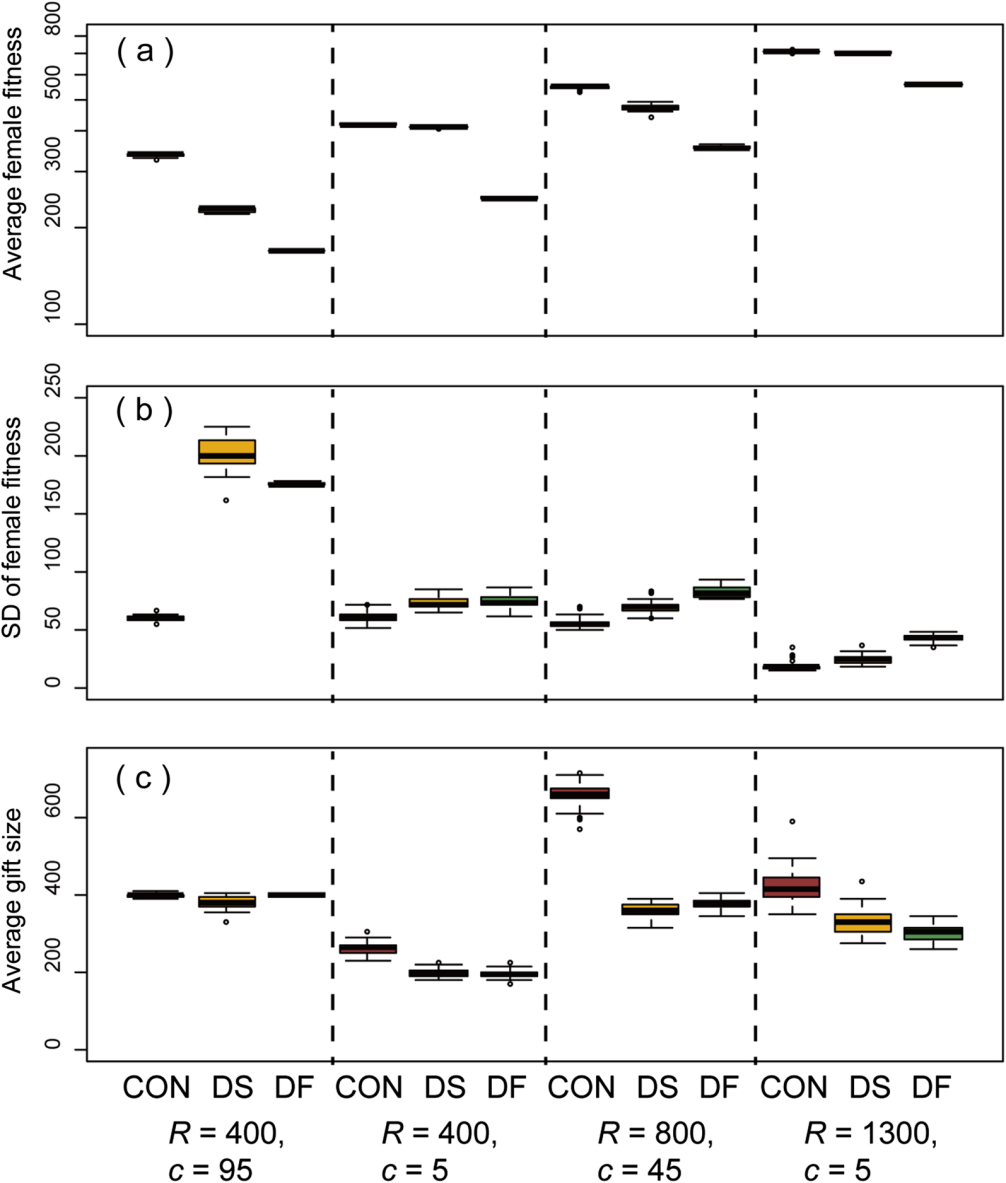
Box plots of the average female fitness **(a)**, SD of female fitness **(b)**, and average male gift size **(c)** observed at the 2000th generation under the FR regime. Results for four different parameter sets, indicated by the asterisks in Fig. 5A-b and the three different simulation modes (CON, control; DS, twin-slots invasion; DF, double the number of females) are shown

Under these conditions, *2s* genes for making the twin slots usually increased rapidly and became fixed (being 95% or more) in the population (Fig. 3a, b). Prevalence of *2S* females caused a coevolutionary reduction in male gift size (Fig. 3a, b). Accordingly, females needed an increased number of matings to approach their fitness optimum, resulting in a reduction in their average fitness (Fig. 6). When mating cost was relatively low, females could not satisfy their demands even with an increase in mating frequency by possessing two slots. However, with a higher mating cost and especially in eutrophic habitats, the smaller sized gifts, as a male counter-adaptation to twin slots, became unattractive to the females, resulting in a higher satisfaction rate (compare Fig. 5A-c with 5A-a).

Strikingly similar patterns were observed when the number of females was doubled at the middle of simulation runs (Fig. 6a; Additional file 1). However, under each parameter set, the observed reduction in fitness was less prominent when *2S* females were fixed compared to when the female number was doubled (Fig. 6a), while among-individual variations in female fitness (measured as SDs) were comparable, being higher than the controls (Fig. 6b; see also Additional file 3).

## Discussion

### Evolution of nuptial gifts: relevance to empirical studies

Our simulation revealed that when mating costs for females are extremely low in oligotrophic environments (low *R* plus low *c*), females cannot satisfy their demands for male derived nutrients regardless of the paternity determination regimes (Fig. 5a). This result supports the view that scarcity of other nutritive sources for female reproduction can be a favorable factor for the evolution of high dependency on male-derived gifts [16, 30]. The paternity-determination regimes also show complicated interactions with these environmental/ecological factors in determining the coevolutionary feedbacks. Except for “low *R* plus low *c*” conditions discussed above, both female traits, high mating rates and twin-slots, rarely evolved in the ES (equal shares) and FM (complete first male) regimes. In the LM (complete last male) regime, males evolved gigantic gifts almost exclusively when their donation results in monandry, supporting the view that male control over female remating rate may be responsible for the origin of nutritious ejaculates in some cases [10, 31]. A high positive correlation between male seminal expenditure and the resultant paternity is a requisite for the evolution of effective nuptial gifts coupled with female polyandry for obtaining the gifts under a wide variety of *R* and *c* (the fair raffle [FR] regime; Fig. 5).

These results provide important insights into the difficulty in discriminating the “mating effort” and “paternal investment” hypotheses (see “Background”). Nutritious nuptial gifts are effective as male mating efforts when females compete to accept (additional) matings to obtain them. The present study revealed that such effective nuptial gifts evolve almost exclusively when donation of large-sized gifts results in high paternity gains, measured as the number of offspring nourished by the female (the FR or LM regime). In these cases, nourishing females as mating efforts by males inevitably results in a higher paternal investment on average.

Although male paternity share is unknown for most animal taxa with nuptial gifts at present, positive correlation between gift size and male paternity share (or the number of sperm stored) has been reported for several cases [13, 15, 32–35]. Among them, in the hangingfly *Hylobittacus apicalis* (Mecoptera: Bittacidae), females accept mating only while eating a nuptial gift (small arthropod prey) donated by the male. Their narrow and elongated spermathecal duct disturbs rapid sperm transfer from males. Therefore, only males who offer a large prey item are allowed to transfer enough sperm to assure their paternity [36]. Similar morphology has been reported for some other insect taxa with nuptial gifts [37, 38].

To keep our model simple, we did not incorporate the following pivotal factors that are known to affect trading of nuptial gifts: assessment of gift size by females before receiving the gift (and deception of gift size by male donors), strategic modulation of gift size by males based on mating status of mates (e.g., unmated vs. previously mated females), and costs of mating itself for males [reviewed in 1,2,5,13,32]. If a large-sized gift attracts more females, exaggeration in gift size may also occur under almost complete first male sperm precedence. For *Neotrogla* spp., in which copulation with the female positioned above the male lasts for a long period (see “Background”), male mating costs can be especially relevant. In addition, seminal gift size can also vary interspecifically through possible macro-evolutionary trade-offs with other male and female traits: donation of anti-aphrodisiac components to females in crickets [39], male calling frequency in bushcrickets [40], male weaponry in megalopteran insects [41], and male bioluminescent courtship and female flightlessness in fireflies [33, 42–44]. These complexities should be taken into account when comparing theoretical predictions with empirical data.

### Evolution of female morphology for gift-acquisition

Like all other members of the tribe Sensitibillini known to date, *Neotrogla* spp. exclusively occur in oligotrophic, dry cave habitats (low *R*: [45, 46]). In addition, they retain ancestral “female above male” mating positions (Fig. 2), suggesting that males do not impose high mating costs on females (low *c*). However, they are apparently polyandrous as evidenced by multiple emptied spermatophore capsules, the volume of which corresponds to ~ 300 ml if scaled up to human proportions [23], in their spermatheca [22]. In our model, such a combination of female multiple mating and large-sized gifts occurred only in the fair raffle (FR) regime (Fig. 4). Though sperm usage/storage patterns are completely unknown at present for the members of Sensitibillini, their spermathecal duct (sperm corridor) is especially elongated and coiled among Psocodea (booklice, barklice, and parasitic lice) [24, 46–50]. Thus, it is plausible that the narrow and extremely long sperm corridor of Sensitibillini also functions to establish a positive correlation between seminal gift size and transferred sperm number (FR regime). Since many psocids show continuous production and deposition of eggs after sexual maturity [51, 52], the LM (complete last male) regime is also a candidate if females mate with a single male in each oviposition interval. Although some *Neotrogla* females possess two freshly deposited spermatophores attached to the twin-slots [22], it is presently unknown whether they are derived from two different males. Future studies should clarify sperm usage patterns in this unique group of insects.

Our simulations also demonstrated that twin slots for obtaining gifts in rapid succession are advantageous when females cannot achieve their required number of matings, and that the evolution of this persistence trait has notable effects on the coevolutionary dynamics in trading of nuptial gifts between the sexes. Little is known about the transitional process from the single-slot state, which certainly is ancestral in psocids, to the twin slots observed uniquely in Sensitibillini. For simplicity, we assumed that single-slot and twin-slots states can be switched by two alleles of a single locus. However, since the spermathecal plate of the extant Sensitibillini is a complex structure harboring not only the twin slots for seminal gifts, but also a muscle-driven mechanism to switch seminal flows between them [23; Fig. 3c], it has likely evolved gradually from primitive precursors during its long evolutionary history of approximately 50 million years (from 177.5 Mya to 127.2 Mya: [53]).

Unlike doubling the female number, the evolution of twin slots usually resulted in less pronounced reduction in female fitness with its comparatively large variance (Fig. 6). A larger variation in the female fitness means stronger sexual selection operating among them. This intensified female-female competition for male-derived nutrients, caused by the evolution of twin slots, could also be a crucial factor favoring the evolution of a manipulative intromittent organ in the female Sensitibillini. Interestingly, detailed comparative morphology and molecular phylogeny of Sensitibillini indicated that a female penis with an intromittent function has evolved twice independently, in *Neotrogla* and *Afrotrogla*, in this small insect tribe [28, 45, 54], although the detailed genital functions of *Afrotrogla* are still unknown.

As a coevolutionary response for filling the increased number of slots, males reduce the size of each gift, resulting in an increase in the optimal number of matings for females. Persistence traits, such as genital spines for anchoring unwilling mates or intromittent organs for traumatic insemination, usually develop in males, and can reduce the total fitness of their mates [55, 56]. Theoretical studies show that this kind of inconsistency between male and female interests can even result in a high risk of extinction, driven by the evolution of male “selfish” traits for escalated male-male competition for mates [57]. This type of trait can also be exaggerated through arms races between the sexes so that females also develop counter-adaptations to resist or tolerate male persistence (sexually antagonistic coevolution; e.g., [58, 59]). The results of the present study clearly indicate that even “persistence” traits can be in the category of sex-reversed traits, driven by the evolution of effective nuptial gifts.

### Evolution of nuptial gifts: relevance to previous models

There are only a few preceding studies on the coevolutionary feedbacks between the sexes in trading of ejaculate components. Like nutritious materials in nuptial gifts, sperm itself can be considered as a limited resource for both sexes [60–68]. Considering a situation equivalent to the FR (fair raffle) regime of this study, a previous study examined the effects of several environmental factors on the evolutionary feedbacks between male sperm allocation strategies and female mating rate [29]. In the model, increased mating costs for females (*c*_*f*_, equivalent to our *c*) resulted in a lower female mating rate and an increased sperm package size, similar to the results of the present study. The study also showed that reduction in resource availability to males (low *R*) results in a monotonic reduction in the ejaculate size [29]. Although our present model showed similar dependency of seminal gift size (*V*) on *R*, prudently allocated gifts were also observed in extremely eutrophic environments with high female mating costs (Fig. 4A). This difference can be attributed to the different assumptions adopted: in the model of Abe and Kamimura [29], a size-number trade-off in seminal production was assumed only when females cannot satisfy their demands for sperm supply.

Bocedi and Reid [69] also examined the effects of varying female mating costs on the coevolutionary feedbacks between male sperm traits (sperm number and sperm longevity) and female mating frequency. Under fair raffle sperm competition, an increase in female mating costs resulted in reduced polyandry, as seen in the present study, but also in a reduction of sperm number transferred during a single mating event [69]. The latter finding makes a striking contrast to our results in which males prepare larger gifts for less polyandrous females unless the environment is not extremely eutrophic (Fig. 4A). This can also be attributed to the different assumptions adopted for delineating trade-off relationships: a trade-off between sperm number and sperm longevity was incorporated in Bocedi and Reid [69] instead of a size-number trade-off. Males should invest more resources to sperm longevity, rather than sperm number, when they experience a low sperm competition risk in less polyandrous females.

## Conclusion

The present study has clearly demonstrated that sperm usage patterns, which have rarely been examined for animals with nuptial gifts, can be a pivotal factor for determining the extent of exaggeration in nuptial gifting. Our simulation results have shown that female multiple matings for obtaining male-derived gifts always evolve under oligotrophic environments with low mating costs. However, exaggeration in gift size occurred only under limited conditions: (1) when gift size is positively correlated with siring success, or (2) when donation of a large gift imposes monandry under high last-male sperm precedence. When females could not achieve their optimal number of matings, a persistent female trait for competitively acquiring gifts can further invade. However, through coevolutionary reduction in male gift size, fixation of this trait can drastically reduce the average female fitness (i.e., population productivity), as is the case with sexually persistence traits in males.

## Methods

### Model assumptions

All notations and parameter values used in this article are summarized in Table 1. It is likely that a given volume of nuptial gift will increase the female’s fecundity more effectively when the female is starving than when she has already received a large amount of nutrients from the preceding mates. Thus, we assumed that the potential offspring number of a female (fecundity, *F*_F_pot_) is a saturation function of the cumulative volume of seminal gifts (*r*) received in all of her previous matings (Fig. 1):

1 $$F_{{{\text{F}}\_{\text{pot}}}} \left( r \right) = F_{{{\text{F}}\_{\text{max}}}} \left( {1 - e^{ - br} } \right)$$

where *b*, set at 0.002 throughout the present study, represents the speed of saturation. In this equation, *F*_F_max_, set at 800, denotes the maximum number of offspring that a female can potentially produce when *r* = ∞ under no cost of mating.

Even in cases in which males donate nuptial gifts at each mating event, multiple mating, which may involve costly mate-searching and an enhanced risk of being predated, can be detrimental for the females. Thus, we imposed a cost (*c*) for each mating event, as a reduction in the number of offspring as:

2 $$F_{{\text{F}}} \left( {r, c, M_{R} } \right) = F_{{{\text{F}}\_{\text{pot}}}} \left( r \right) - c \cdot M_{R}$$

where *M*_*R*_ is the number of times a focal female mates. Since we assumed no differential mortality between genotypes in this study, the fecundity delineated by Eq. 2 is directly proportional to the female fitness, i.e., the number of reproductives of the next generation. Thus, Eq. 2, which represents the realized fecundity, was used as the expected fitness of females.

This simple function delineates complicated coevolutionary relationships between the male and female traits (Fig. 1). When males donate a large-sized gift at each mating (e.g., *V* = 500), an additional mating further increases the lifetime fitness of a singly-mated female under a low-cost mating (e.g., *c* = 100; orange arrows in Fig. 1), but not when it is largely costly (e.g., *c* = 200; red arrows).

We assumed that reception of a gift, which occupy a slot for its digestion, delays subsequent matings of the female, in a manner proportional to the gift size. Thus, from a male perspective, males that produce large-sized gifts can reduce the probability that a female remates while increasing their fecundity. However, given a limited amount of resource available for gift production (*R*), males with large *V* suffer a reduced number of possible matings (*N* ≈ *R*/*V*; a size-number trade-off).

We examined four different regimes for the relationship between the nuptial gift size and the resultant paternity gain in this study: (1) fair raffle (FR), (2) equal shares (ES), (3) complete last male (LM), and (4) complete first male (FM) (Fig. 2, Table 1). In the case that males can transfer more sperm by giving a large-sized gift (the FR regime), it can result in a higher paternity share compared to males who gave a smaller gift to the same mate.

Given these assumptions, the expected fitness of a male (*F*_M_) that donated a total of *n* (*n* ≤ *N*) gifts to females can be written as:

3 $$F_{{\text{M}}} \left( V \right) = \mathop \sum \limits_{i = 1}^{n} P_{i} F_{{{\text{F}}i}}$$

where *P*_*i*_ and *F*_Fi_ are the paternity share and the realized fecundity (= expected fitness given by Eq. 2) of the female that received the *i*th gift from the focal male. Under the ES (equal shares) paternity determination regime, *P*_*i*_ is 1/*M*_*R*_ if the female mate *M*_*R*_ times before offspring production. It is 1 (0) or 0 (1) when the focal male is the first (last) male of a mate or not, under the complete first male (FM) (complete last male [LM]) regime, respectively. However, the probability for being the first (or last) male depends on both the gift size of the focal male (*V*_focal_) and those of *M*_*R*_ rival males, requiring an individual-based simulation approach for solving the coevolutionary dynamics. It is also true for the fair raffle (FR) regime, in which *P*_*i*_ is given as:

4 $$P_{i} = V_{{{\text{focal}}}} /\mathop \sum \limits_{j = 1}^{{M_{R} }} V_{j} = V_{{{\text{focal}}}} /r$$

### Simulation details

Individual-based simulations were conducted to observe the coevolutionary dynamics of three traits: male seminal gift size, female propensity for multiple mating, and the number of slots for obtaining gifts in females. For this, we used a personal script written in Python 3.7.1, which is provided as Additional file 4. Simulations were run for 2000 generations assuming a single population of diploid organisms with sexual reproduction and discrete generations. The population size was set at 1000 (500 males and 500 females). Nuptial gift size, a trait of male-specific expression, was assumed to be determined by many alleles. To mimic sexual reproduction, we assumed a single locus with infinitely many possible alleles for this trait (continuum of alleles model). The initial value for each gene was randomly extracted from a normal distribution, with a mean of 20 and a standard deviation (SD) of 40. Each individual possesses two values, *v1* and *v2*, as the genotype of this trait. The seminal gift size (volume; *V*) was determined as (*v1* + *v2*)/2 only for male individuals as their phenotype. Similarly, a mean of 0.5 ± 2 was given as the initial values for the propensity for multiple mating (*m1* and *m2*), which determines the maximum number of “additional” matings accepted by each female individual (*M*), as the nearest integer of (*m1* + *m2*)/2. Thus, females of the genotype *m1*/*m2* mate *M* + 1 times, whenever a mating opportunity is available. To prevent the occurrence of unreasonable values in these two traits, we set the lower limits of genetic values of these two traits as 20 and 0, respectively.

Another female trait, the number of slots for accepting nuptial gifts, was assumed to be a dichotomous trait, that is, one or two slots. Little is known about the evolutionary process of the spermathecal plate, which enables retention of two nuptial gifts simultaneously (see "Discussion”). For simplicity, we assumed that the transition is controlled by a single locus with two alleles: *2s*, a gene for the twin-slot state, and *1s*, a gene for the single-slot state, the former was assumed to be dominant over the latter. The populations were initiated with single-slot females (*1S*, *1s*/*1s* homozygotes). Then, *2s* genes were allowed to invade to populations at the 1000th generation and beyond (one spontaneous mutation in each generation), together with recurrent invasion of single-slot mutants (a female individual of *1s*/*1s* homozygote per generation) to check the evolutionary stability of the twin-slot state (*2S*). When the *2S* phenotype occupied 95% or more of females, this phenotype was judged as fixed.

In the model, each male can mate up to *N* times, which is *R*/*V* rounded up to the nearest integer, where *R* is a total volume of resource available for production of nuptial gifts, randomly extracted from a normal distribution. The gift size of the last mating (*V*_*last*_) is:

5 $$V_{last} = R - \left( {N - 1} \right)V$$

The mean value of *R* ranged from 400 to 1300, by increments of 100, representing an environmental variability in resource availability (oligotrophic to eutrophic). The SD was set at each mean value multiplied by 0.2.

In our model, we considered only the effects of post-copulatory sexual selection on the coevolution between male gift size and female traits for obtaining gifts. Any precopulatory behaviors, such as male-male combats for mates or female choice on gift size or males, were not incorporated. For implementing this condition, seminal gifts produced by all male individuals were pooled and considered as mating opportunities in every generation. Males producing many small gifts represent a larger proportion of this “gift pool”, incorporating the size-number trade-off specified above. We assumed that all virgin females start to mate simultaneously, by randomly assigning a gift from the pool to a slot. This procedure guarantees that all females mate at least once, unless the sex ratio is female-biased (doubled: see below) and males produce only a few gifts (less than two on average) per capita. A female accepts additional mating when (1) she has mated fewer times than her acceptable number of matings (*M* + 1), and (2) at least one of her slots are empty. Remaining seminal gifts in the pool were then sequentially assigned, one at a time, to the slot that had received the minimum cumulative volume of gifts (*r*) in females whose mating demand was not satisfied at the time. Thus, reception of a large gift, which is difficult to digest for females, resulted in delayed remating of the female. This procedure was repeated until all females satisfied their mating demands, or the gift pool became empty (= all males exhausted their resource budgets).

Then, the expected fecundity of each female (*F*) was calculated according to Eq. 2. Cost of mating, defined by a reduction in female fitness per mating (Fig. 1), was varied from 5 to 95, by increments of 10, and negative fitness values were treated as zero. To keep the constant population size, maternity and paternity of offspring were determined stochastically according to the proportional representation of the relative fitness of females (Eq. 2) and the relative representation of male sperm in each female (*P*_*i*_ of Eq. 3), respectively. For each trait, one of two parental genes was randomly and independently extracted from two parents, and fused to create an individual of the next generation, that is, with no linkage among the three traits. For seminal gift size and propensity for female multiple mating, each of these values was treated as the mean of a normal distribution for creating the genetic values of progenies (recurrent mutation) with SDs 40 or 2, respectively.

To dissect the complicated coevolutionary interactions between the male and female traits, simulations were repeated 40 times for each combination of *R* and *c* values, and for each of the four different paternity determination regimes (FR, ES, LM, and FM) specified above (Fig. 2). In addition, three different types of simulations were conducted (Table 1). Abe and Kamimura [29] demonstrated that female-biased sex ratios promote the production of smaller ejaculate packages by males in order to mate with more females. Doubling the slot number can have a similar effect on male ejaculate allocation. For comparison with doubling-slot (DS) runs, in which one twin-slot mutant female (2S female) was introduced every generation after the 1000th generation, the number of females was doubled, resulting in 1000 females per 500 males, from the 1000th generation and beyond in doubling females (DF) runs. In the control runs (CON), neither the numbers of slots nor females were doubled throughout the 2000-generation runs. Possible effects of genetic correlation between the male and female traits on their coevolution were also examined (see Additional file 2).

## Supplementary Information

**Additional file 1.** Examples of the coevolutionary dynamics between the male seminal gift size and optimal number of matings for females observed in CON (control) and DF (doubling females) runs.

**Additional file 2.** Examination of genetic correlation between the male seminal gift size and optimal number of matings for females.

**Additional file 3.** Comparisons of relative intensity of sexual selection between the sexes.

**Additional file 4.** Model source code written in Python 3.7.1.

## Data Availability

All data was generated by the Pyhton script, which is provided as Additional file 4. All data is deposited in in the Dryad Digital Repository: https://doi.org/10.5061/dryad.n5tb2rbtc.
