# Supplementary material for: Evolution of nuptial gifts and its coevolutionary dynamics with male-like persistence traits of females for multiple mating
Source: BMC Ecol Evol. 2021 Sep 5;21:164. doi: 10.1186/s12862-021-01901-x (PMC8419916; doi:10.1186/s12862-021-01901-x)
Supplement: Supplementary file 1 — Additional file 1. Examples of the coevolutionary dynamics between the male seminal gift size and optimal number of matings for females observed in CON (control) and DF (doubling females) runs. [file 12862_2021_1901_MOESM1_ESM.pdf]

ADDITIONAL FILE 1 for "Evolution of nuptial gifts and its coevolutionary dynamics with male-like persistence traits of females for multiple mating."

In the present study, we conducted three different types of simulations: in doubling the number of females (DF) runs, the number of females was doubled, resulting in 1000 females per 500 males, from the 1000th generation and beyond, instead of introducing invasion of one twin-slot mutant female (*2S* female) every generation (doubling slots: DS runs). In the control runs (CON), neither the numbers of slots nor females were doubled throughout the 2000-generation runs. For comparison with example results of the DS runs shown in Figure 3 of the main text, Figure S1.1 shows the results of CON and DF runs under the same parameter values.

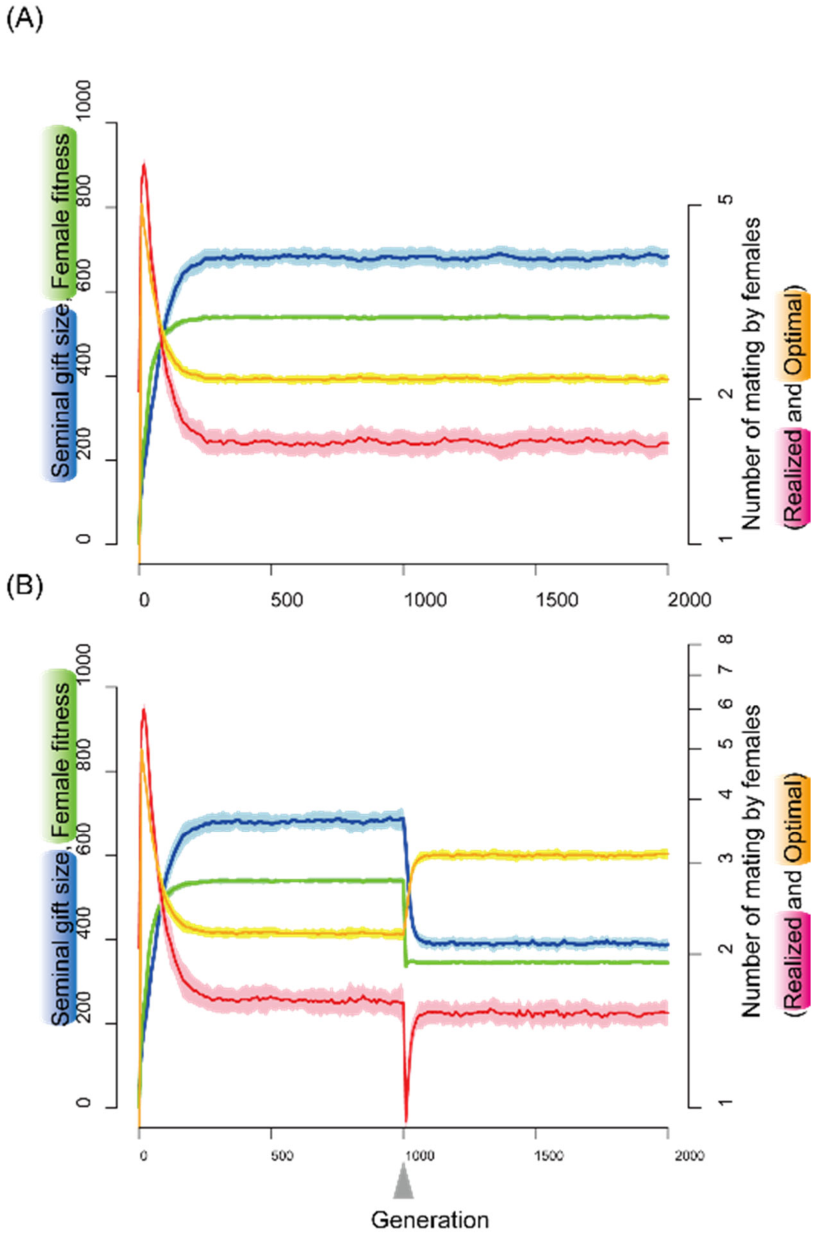

Figure S1.1 **(A)** An example of the coevolutionary dynamics between the male seminal gift size (*V*, blue) and optimal number of matings for females (M_O, red) observed in CON runs, together with changes in the realized number of matings by females (M_R, orange) and female fitness (*F*, green). Solid lines and shaded areas of respective lighter colors show the mean ± SD for 40 runs under the FR regime (*R* = 800, *c* = 55). **(B)** An example of the coevolutionary dynamics observed in DF runs: the number of females was doubled, from 500 to 1000, at the 1000th generation (indicated by the grey arrowhead). Other conditions and notations are same as **(A)**.
