# Supplementary material for: Evolution of nuptial gifts and its coevolutionary dynamics with male-like persistence traits of females for multiple mating
Source: BMC Ecol Evol. 2021 Sep 5;21:164. doi: 10.1186/s12862-021-01901-x (PMC8419916; doi:10.1186/s12862-021-01901-x)
Supplement: Supplementary file 2 — Additional file 2. Examination of genetic correlation between the male seminal gift size and optimal number of matings for females. [file 12862_2021_1901_MOESM2_ESM.pdf]

ADDITIONAL FILE 2 for "Evolution of nuptial gifts and its coevolutionary dynamics with male-like persistence traits of females for multiple mating."

Exaggerated male traits (such as ornaments for attracting females) and female preference for them can coevolve through two different mechanisms of sexual selection, namely, an increase in the number of offspring (direct benefits) or enhanced genetic quality of the offspring (indirect or genetic benefits) for a female who executed mate choice [1]. In other words, a genetic association between the male ornamental traits and female preference for them can drive self-reinforcing coevolutionary feedbacks among them (Fisherian runaway process; [2]). As preferences for specific male traits, female propensity for polyandry can also evolve for increased offspring quality (e.g., [3]).

To evaluate its effects on the coevolutionary feedbacks, the genetic correlation coefficient between the genetic values of male nuptial gift size ([*v1* + *v2*] / 2) and the propensity for multiple mating in females ([*m1*+ *m2*] / 2) was recorded in the simulations. In addition, by shuffling paternally-derived genes (= randomly resampling without replacement) among all offspring of the population in each generation, possible genetic correlations between the two traits were eliminated in supplemental trials.

As the results, the genetic correlation was negligibly low at near zero (Figure S2.1A), and thus elimination of genetic covariance by shuffling paternal identity did not change the results (Figures S2.1B, S2.2, S2.3). These results indicate that the observed coevolutionary dynamics are mainly driven by "direct" benefits of mating for both males and females.

Literature Cited in ADDITIONAL FILE 2

1. Andersson M. Sexual selection. Princeton, NJ: Princeton University Press; 1994.
2. Fisher RA The genetical theory of natural selection. Oxford: Clarendon Press; 1930.
3. Yasui Y. A 'good-sperm' model can explain the evolution of costly multiple mating by females. Am Naturalist. 1997; 149:573–584.

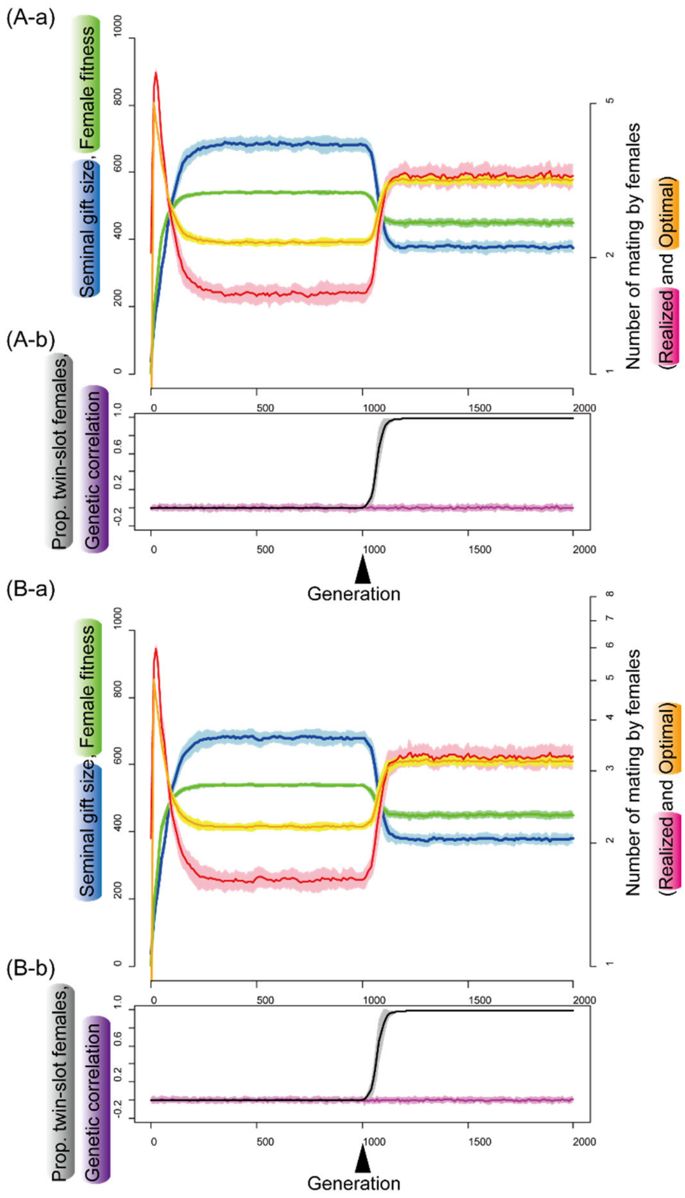

Figure S2.1 (A-a) An example of the coevolutionary dynamics observed between the male seminal gift size (V, blue) and optimal number of matings for females (M_O, red), together with changes in the realized number of matings by females (M_R, orange) and female fitness (F, green). **(A-b)** Changes in the proportion of females with twin slots ($2S$, black), which started to invade the population at the 1000th generation (the black arrowhead), and the correlation coefficient between genetic values of seminal gift size and female mating propensity (purple). Solid lines and shaded areas of respective lighter colors show the mean ± SD for 40 runs under the FR regime (R = 800, c = 55). **(B-a)** and **(B-b)** are the same as **(A-a)** and **(A-b)**, respectively, but the genetic correlation was eliminated by shuffling paternal identity of offspring.

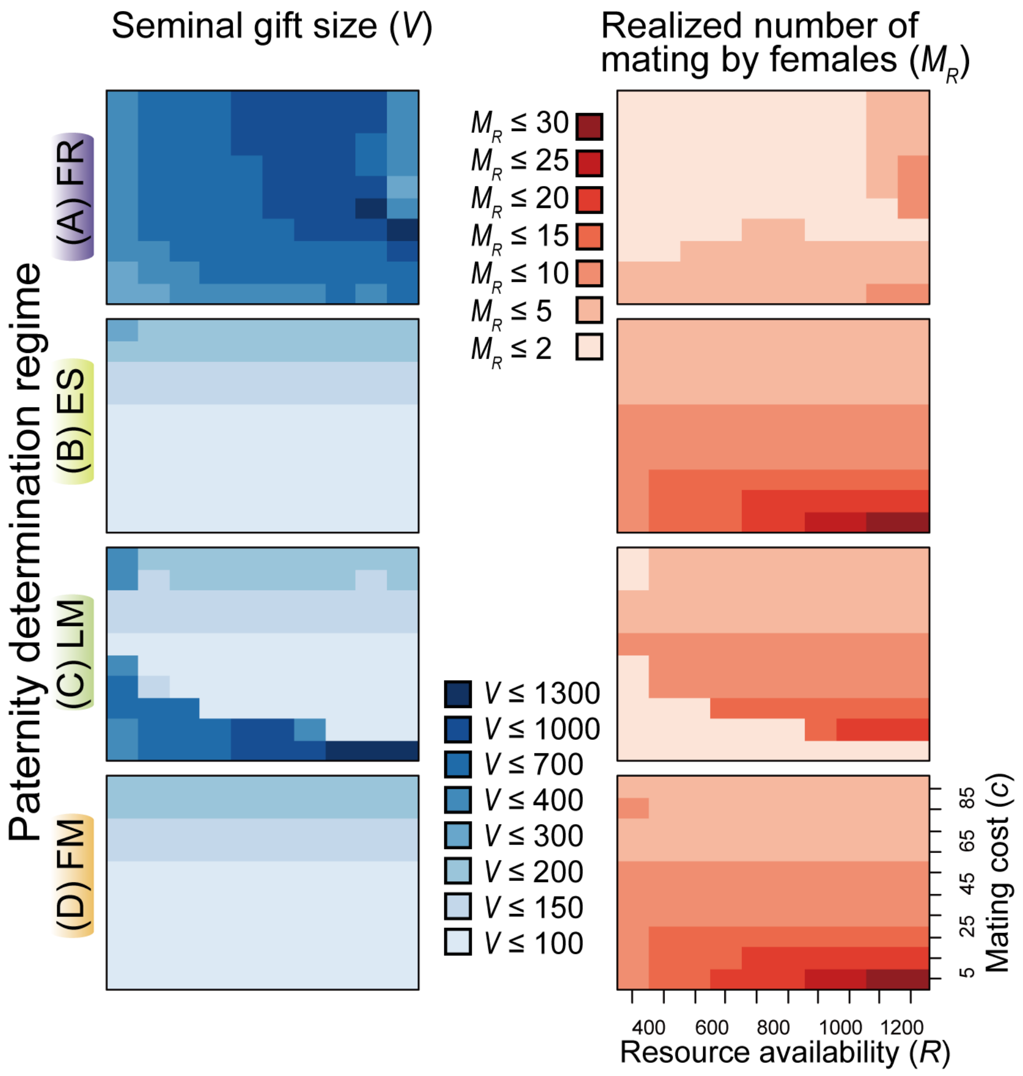

Figure S2.2 Average male seminal gift size (*V*, light blue backgrounds) and the realized number of matings by females (*MR*, pink backgrounds) at the 2000th generation observed in the control runs (CON) of four different paternity-determination regimes (**A,** FR; **B,** ES; **C,** LM; and **D,** FM). The conditions of the simulation were identical to those of **Figure. 4** of the main text, but the genetic correlation was eliminated by shuffling paternal identity of offspring.

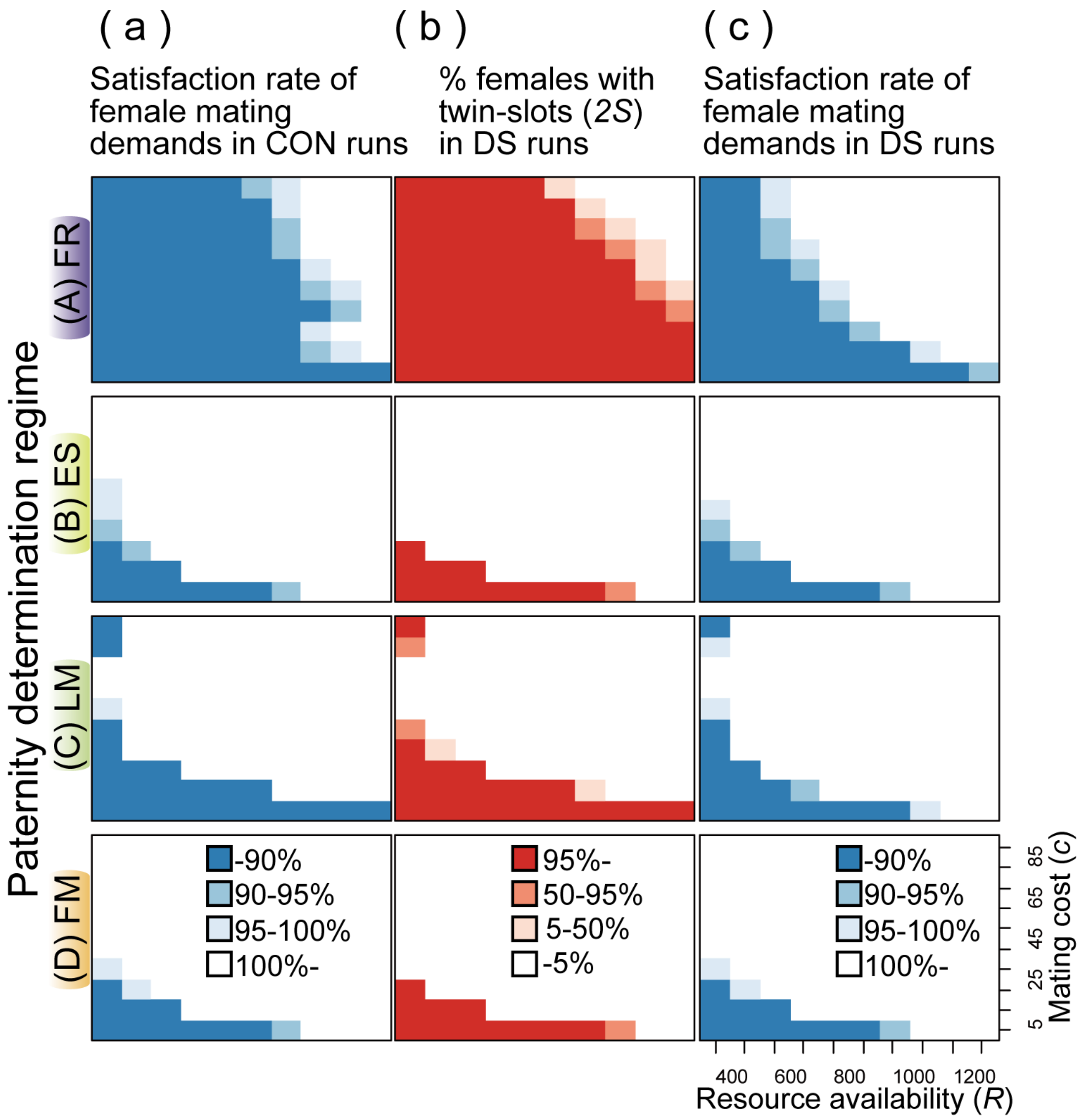

Figure S2.3 Average satisfaction rate (*MR* / *MO*) of female mating demands in CON **(a)** and DS **(c)** runs, and proportion of *2S* females **(b)** in DS runs at the 2000th generation (**A,** FR; **B,** ES; **C,** LM; and **D,** FM). The conditions of the simulation were identical to those of **Figure. 5** of the main text, but the genetic correlation was eliminated by shuffling paternal identity of offspring.
