# Supplementary material for: Evolution of nuptial gifts and its coevolutionary dynamics with male-like persistence traits of females for multiple mating
Source: BMC Ecol Evol. 2021 Sep 5;21:164. doi: 10.1186/s12862-021-01901-x (PMC8419916; doi:10.1186/s12862-021-01901-x)
Supplement: Supplementary file 3 — Additional file 3. Comparisons of relative intensity of sexual selection between the sexes. [file 12862_2021_1901_MOESM3_ESM.pdf]

ADDITIONAL FILE 3 for "Evolution of nuptial gifts and its coevolutionary dynamics with male-like persistence traits of females for multiple mating."

Although it is difficult to quantify selection pressure in wild populations, sexual selection is considered to exert a stronger effect in females than males in sex-role reversed animals (e.g., [1]). Female persistence traits, such as doubled slots that have evolved so that they could receive more gifts from males, likely accelerate female-female competition for mates, thus resulting in stronger sexual selection among females.

The intensity of selection is usually measured in terms of variance in fitness. Figure S3.1a shows the relative intensity of sexual selection given by the SD of the actual female fitness divided by the SD of the actual male fitness, for four different parameter sets under the FR regime (as in Figure 6).

Since female reproductive output is completely dependent on male-derived nutrients in the present model, variation in female fitness was usually comparable to or larger than that of males, unless the sex-ratio is strongly female-biased (DF runs). Analogous to an increased harem size in polygynous mating systems (Wade and Shuster 2004), the "fewer large" strategy of nuptial gifts likely increased the variance in male or female fitness when the reproductive success is highly skewed in the respective sex. The notably strong sexual selection in females in DS runs under a low R (400; oligotrophic) and high c (95; because of this high mating cost, females have to gather nutrients from a small number of matings) is likely caused by a combination of the "fewer large" strategy adopted by the males and the evolution of twin slots in females.

Literature Cited in ADDITIONAL FILE 3

1. Kamimura Y, Yoshizawa K. Sex role reversal. In: Vonk J, Shackelford TK, editors. Encyclopedia of Animal Cognition and Behavior. Berlin: Springer International Pub.; 2017. DOI:10.1007/978-3-319-47829-6_2012-1.
2. Wade MJ, Shuster SM. Sexual selection harem size and the variance in male reproductive success. Am Naturalist. 2004; 164:E83–E89.

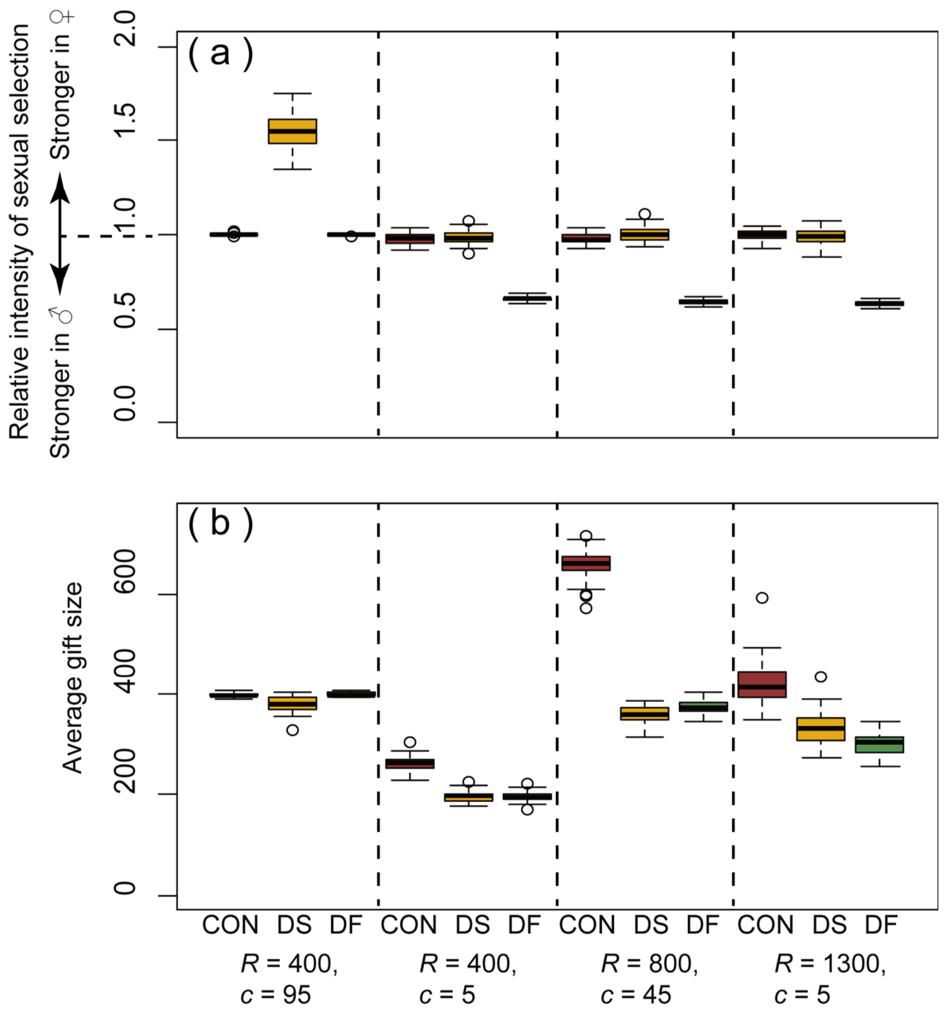

Figure S3.1 Box plots of the relative intensity of sexual selection (a) and average male gift size (b) observed at the 2000th generation under the FR regime. The relative intensity of sexual selection is given by the SD of the actual female fitness divided by the SD of the actual male fitness. The results obtained using four different parameter sets, indicated by the asterisks in Figure 5A-a and the three different simulation modes (CON, control; DS, twin-slots invasion; DF, double the number of females), are shown. Note that instead of the expected fitness (fecundity) determined by eq. 2 (used in Figures 3 and 6), the actual fitness of both sexes (i.e., the number of offspring survived to adulthood) was used to calculate the relative intensity of sexual selection.
