# Supplementary material for: Evolution of nuptial gifts and its coevolutionary dynamics with male-like persistence traits of females for multiple mating
Source: BMC Ecol Evol. 2021 Sep 5;21:164. doi: 10.1186/s12862-021-01901-x (PMC8419916; doi:10.1186/s12862-021-01901-x)
Supplement: Supplementary file 4 — Additional file 4. Model source code written in Python 3.7.1. [file 12862_2021_1901_MOESM4_ESM.pdf]

```
import os
import time
import numpy as np
from numpy.random import * ###For generating random numbers
from numpy import * ### For using "random.choice"

np.random.seed(0)

######DEFINITIONS######
simrun = 40; simgen = 2001; # No. of iterations/generations
printgen = 10; printcount = 0; # Print records every "printgen" generation

invgen = 11000; #Initiation of invation of twin-slots mutants
doublegen = 11000; #Doubling female number at generation "doublegen"

min_sem_vol = 20;

nm = nf = ininf = 500; #Population size (nm, male number; nf, female number)
R = Rini = 400; Rsdratio = 0.2; R_sd = R * Rsdratio; #Average/SD of the total male resource for reproduction
Rin = 100; Rite = 10;
b = 800; #Maximum female reproductive output
k = 0.002; #Fertilization efficiency (this parameter represents the saturating speed of female reproductive output)
mate_cost = 5; #Decrease in female fitness per mating
MCin = 10; MCite = 10;

no_gencor_mode = 0; #Genetic correlation between male and female traits: 0, present; 1, removed
paternity_mode = 0;
#0, fair raffle based on seminal gift size (FR); 1, equal shares (ES); 2, complete last male paternity (LM); 3,
complete first male paternity (FM)

now = time.time();
fmt = '%Y-%m-%d';
filename = time.strftime(fmt, time.localtime(now)) + '_1_run_' + str(simrun) + '_inv_' + str(invgen) + '_dbl_' +
str(doublegen) + '_GCmode_' + str(no_gencor_mode) +'_PATmode_' + str(paternity_mode) + '_limM_' + str(min_sem_vol) +
'-' + '_n_' + str(nm) + '_R(sdratio)_' + str(Rsdratio) + '_Rincre' + '_b_' + str(b) + '_k_' + str(k) +
'Mcostincre.csv';

######Initial genetic values of traits and mutation parameters######
ini_mean_m = 20; #Initial mean of seminal gift size
ini_mean_f = 0.5; #Initial mean of the No. of additional matings by females
```

```
sd_m = 40 # SD of seminal seminal gift size
sd_f = 2; # SD of No. additional mating by females

mute_m = 40; #Mutation (SD) in seminal gift size
mute_f = 2; #Mutation (SD) in No. additional mating

#####MAIN FUNCTION#####
Records = np.empty(int(((simgen/printgen)*simrun*Rite*MCite + simrun*Rite*MCite)) *
18).reshape(int((simgen/printgen)*simrun*Rite*MCite + simrun*Rite*MCite),18);
###"Simrun","Simgen", "Time","Ave_m","SD_m", "Ave_f", "SD_f", "r-Ave_f", "r-SD_f",
"propSppl","Ave_rec_sp","SD_rec_sp", "Ave_fitness", "SD_fitness","opt_mate_num", "gen_cor");

for mcite in range(MCite):
    R = Rini;
    for rite in range(Rite):
        for sr in range(simrun):
            #####SETTING INITIAL POPULATION#####
            nf = ininf
            males = np.vstack((np.random.normal(ini_mean_m, sd_m, nm), np.random.normal(ini_mean_m, sd_m, nm),
np.random.normal(ini_mean_f, sd_f, nm), np.random.normal(ini_mean_f, sd_f, nm), np.zeros(nm), np.zeros(nm)));
            females = np.vstack((np.random.normal(ini_mean_m, sd_m, nf), np.random.normal(ini_mean_m, sd_m, nf),
np.random.normal(ini_mean_f, sd_f, nf), np.random.normal(ini_mean_f, sd_f, nf), np.zeros(nf), np.zeros(nf)));

            nx_males = np.empty(6 * nm).reshape(6,nm); nx_females = np.empty(6 * nf).reshape(6,nf); ####for next
generation

            for N in range(simgen):
                males[0:2,:] = np.where(males[0:2,:] < min_sem_vol, min_sem_vol, males[0:2,:]); females[0:2,:] =
np.where(females[0:2,:] < min_sem_vol, min_sem_vol, females[0:2,:]);###Values < 20 -> 20
                males[2:4,:] = np.where(males[2:4,:] < 0, 0, males[2:4,:]); females[2:4,:] = np.where(females[2:4,:]
< 0, 0, females[2:4,:]);###Negative values -> 0
                if N >= invgen:
                    randint0 = randint(0,nf); randint1 = randint(0,nf);
                    females[4:5,randint0:(randint0 + 1)] = 1;
                    females[4:5,randint1:(randint1 + 1)] = 0; females[5:6,randint1:(randint1 + 1)] = 0;

                sppl = sign(females[[4]]+females[[5]]).reshape(-1); nsppl = sum(sppl).astype(int32); sppl =
sppl.astype(int32) + 1; nslot = nf + nsppl;
                MALES = np.vstack((np.arange(nm), (males[[0]] + males[[1]])/2, np.empty(nm), np.random.normal(R,
```

```
R_sd, nm), np.empty(nm)));
                MALES[[3]] = np.where(MALES[[3]] < min_sem_vol, min_sem_vol, MALES[[3]] ); MALES[[1]] =
np.where(MALES[[1]] > MALES[[3]], MALES[[3]], MALES[[1]] );
                MALES[[2]] = np.ceil(MALES[[3]]/MALES[[1]]); MALES[[4]] = MALES[[3]] - (MALES[[2]]-1) * MALES[[1]];
                f_add_cop = np.round((females[[2]] + females[[3]]) / 2);
                genFmean = np.mean((f_add_cop+1)*sppl); genFstd = np.std((f_add_cop+1)*sppl); ###For recording
female "desired" number of matings
                M_cop = np.arange(nm).repeat(MALES[[2]].astype(int32).reshape(nm));
                M_vol = MALES[[1]].repeat(MALES[[2]].astype(int32).reshape(nm)); M_last_vol =
MALES[[4]].repeat(MALES[[2]].astype(int32).reshape(nm));
                M_COP = np.vstack((M_cop, M_vol, np.empty(len(M_cop)), np.empty(len(M_cop)), M_last_vol));
                ### M_COP: [0] male_no; [1] seminal_volume; [2] slot_no(female);  [3] female_no; [4] seminal volume
of last copulation
                M_COP = M_COP[:,np.argsort(np.random.rand(len(M_cop)))]; ###sort based on rand
                last_cop = np.empty(nm);
                for j in range(nm): last_cop[j] = np.max(np.where(M_COP[[0]] == j));
                for l in last_cop.astype(int32): M_COP[1,l] = M_COP[4,l];
                M_COP = np.delete(M_COP, np.where(M_COP[[1]] < min_sem_vol)[1], axis =1);
                F_COP =
np.vstack((np.arange(nslot),np.arange(nf).repeat(sppl),f_add_cop.repeat(sppl),np.zeros(nslot),np.zeros(nslot),
np.empty(nslot)));
                ###F_COP: [0] slot_no; [1] female_no; [2] add_cop_no (acceptable limit); [3]
cum_seminal_volume_received; [4] no_mating; [5] no_sppl
                F_COP[[5]] = np.hstack((1,np.diff(F_COP[[1]]).reshape(-1))); F_COP[[2]] += 1;
                F_COP_unique = np.delete(F_COP, np.where(F_COP[[5]] == 0)[1], axis =1);
            #####MATING#####
                counter = 0;
                if size(F_COP[[0]]) > 0:
                    for i in range(0, size(M_COP[[0]])):
                        sampled_no = np.argmin(F_COP[[3]]);
                        sampled_slot = F_COP[0,sampled_no].astype(int); sampled_female =
F_COP[1,sampled_no].astype(int);
                        M_COP[2,i] = sampled_slot; M_COP[3,i] = sampled_female;
                        F_COP[2,sampled_no] -= 1; F_COP[3,sampled_no] += M_COP[1,i];
                        if F_COP[2,sampled_no].astype(int) <= 0: F_COP = np.delete(F_COP, np.where(F_COP[[2]] ==
0)[1], axis =1);
                        F_COP_unique[3,sampled_female] += M_COP[1,i]; F_COP_unique[4,sampled_female] += 1;
                        counter += 1;
                        if size(F_COP[[0]]) == 0: break;
            ##### REPRODUCTION #####
```

```
                F_fitness = b * (1 - exp(-1* k * F_COP_unique[[3]])) - (mate_cost * F_COP_unique[[4]]);
###Calculation of female fitness
                F_fitness = np.where(F_fitness<0,0,F_fitness).reshape(-1); mean_F_fit = np.mean(F_fitness);
std_F_fit = np.std(F_fitness);
                F_fitness = F_fitness/np.sum(F_fitness); ###Calculation of "relative" female fitness
                M_COP = M_COP[:, 0:counter];
                if paternity_mode == 0:
                    totals = F_COP_unique[3,M_COP[[3]].astype(int)].reshape(-1); M_COP[[1]] =  M_COP[[1]]/totals;
                elif paternity_mode == 1:
                    totals = F_COP_unique[4,M_COP[[3]].astype(int)].reshape(-1); M_COP[[1]] =  1/totals;
                elif paternity_mode == 2:
                    M_COP = np.fliplr(M_COP); unique_array, index_array = np.unique(M_COP[[3]], return_index=True);
M_COP = np.vstack((M_COP[0,index_array],np.repeat(1,len(index_array)),M_COP[2,index_array],unique_array));
                else:
                    unique_array, index_array = np.unique(M_COP[[3]], return_index=True); M_COP =
np.vstack((M_COP[0,index_array],np.repeat(1,len(index_array)),M_COP[2,index_array],unique_array));
                mates = M_COP[[3]].reshape(-1).astype(int); mate_rel_fitness = F_fitness[mates];
                M_COP[[1]] = M_COP[[1]] * mate_rel_fitness; M_COP[[1]] = np.cumsum(M_COP[[1]]);
                prevnf = nf;
                if N == doublegen: nf = 2 * nf;
                ofsp_f = np.histogram(np.random.rand(nf), bins = np.hstack((0,M_COP[[1]].reshape(-1))))[0];
                ofsp_m = np.histogram(np.random.rand(nm), bins = np.hstack((0,M_COP[[1]].reshape(-1))))[0];
                d_mothers = M_COP[[3]].repeat(ofsp_f).astype(int); ### ID of mothers of daughters
                d_fathers = M_COP[[0]].repeat(ofsp_f).astype(int); ### ID of fathers of daughters
                s_mothers = M_COP[[3]].repeat(ofsp_m).astype(int); ###ID of mothers of sons
                s_fathers = M_COP[[0]].repeat(ofsp_m).astype(int); ###ID of fathers of sons
                if no_gencor_mode == 1:
                    d_fathers = d_fathers[np.argsort(np.random.rand(len(d_fathers)))]; ###sort based on rand
###Randomization of father ID
                    s_fathers = s_fathers[np.argsort(np.random.rand(len(s_fathers)))]; ###sort based on rand  ###for
removing genetic correlation
            #####RECORDING RESULTS#####
                if N % printgen == 0:
                    sizeMmean = np.mean(MALES[[1]]); sizeMstd = np.std(MALES[[1]]);
                    opt_num_mate = np.log(mate_cost/(b*k*sizeMmean))/(-k*sizeMmean);
                    gencor = np.corrcoef(hstack(((males[[0]] + males[[1]])/2,(females[[0]] + females[[1]])/2)),
hstack(((males[[2]] + males[[3]])/2,(females[[2]] + females[[3]])/2)))[0,1]
                    Records[[printcount]] = np.hstack((sr, N, time.time()-now, R, mate_cost,sizeMmean, sizeMstd,
genFmean, genFstd, np.mean(F_COP_unique[[4]]),np.std(F_COP_unique[[4]]),((nslot - prevnf)/prevnf),
np.mean(F_COP_unique[[3]]), np.std(F_COP_unique[[3]]), mean_F_fit, std_F_fit, opt_num_mate, gencor));
```

```
                    printcount += 1;
            ##### INHERITANCE #####
                if prevnf != nf: nx_females = np.empty(nf*6).reshape(6,nf);
                swit_m = binomial(1, 0.5, (nm * 6)).reshape(6, nm); swit_f = binomial(1, 0.5, (nf * 6)).reshape(6,
nf);
                nx_males[0] = swit_m[[0]]*males[[0],[s_fathers]] - (swit_m[[0]]-1)*males[[1],[s_fathers]]
                nx_males[1] = swit_m[[1]]*females[[0],[s_mothers]] - (swit_m[[1]]-1)*females[[1],[s_mothers]]
                nx_males[2] = swit_m[[2]]*males[[2],[s_fathers]] - (swit_m[[2]]-1)*males[[3],[s_fathers]]
                nx_males[3] = swit_m[[3]]*females[[2],[s_mothers]] - (swit_m[[3]]-1)*females[[3],[s_mothers]]
                nx_males[4] = swit_m[[4]]*males[[4],[s_fathers]] - (swit_m[[4]]-1)*males[[5],[s_fathers]]
                nx_males[5] = swit_m[[5]]*females[[4],[s_mothers]] - (swit_m[[5]]-1)*females[[5],[s_mothers]]
                nx_females[0] = swit_f[[0]]*males[[0],[d_fathers]] - (swit_f[[0]]-1)*males[[1],[d_fathers]]
                nx_females[1] = swit_f[[1]]*females[[0],[d_mothers]] - (swit_f[[1]]-1)*females[[1],[d_mothers]]
                nx_females[2] = swit_f[[2]]*males[[2],[d_fathers]] - (swit_f[[2]]-1)*males[[3],[d_fathers]]
                nx_females[3] = swit_f[[3]]*females[[2],[d_mothers]] - (swit_f[[3]]-1)*females[[3],[d_mothers]]
                nx_females[4] = swit_f[[4]]*males[[4],[d_fathers]] - (swit_f[[4]]-1)*males[[5],[d_fathers]]
                nx_females[5] = swit_f[[5]]*females[[4],[d_mothers]] - (swit_f[[5]]-1)*females[[5],[d_mothers]]
            ##### MUTATION -> TO NEXT GENERATION #####
                if prevnf != nf: females = np.empty(nf*6).reshape(6,nf);
                males[[0,1]] = np.random.normal(nx_males[[0,1]], mute_m); females[[0,1]] =
np.random.normal(nx_females[[0,1]], mute_m);
                males[[2,3]] = np.random.normal(nx_males[[2,3]], mute_f);females[[2,3]] =
np.random.normal(nx_females[[2,3]], mute_f);
                males[[4,5]] = nx_males[[4,5]]; females[[4,5]] = nx_females[[4,5]];
                females = females[:,np.argsort(np.random.rand(nf))];###End of "simrun" loop;
        R += Rin
    mate_cost += MCin

now2 = time.time();print((now2-now)/60);
np.savetxt(filename, Records, fmt ='%.2f', delimiter = ',');
```
